# Parkinson’s Disease-Cognitive Rating Scale for Evaluating Cognitive Impairment in Parkinson’s Disease: A Systematic Review

**DOI:** 10.3390/brainsci10090588

**Published:** 2020-08-25

**Authors:** Elena Cecilia Rosca, Mihaela Simu

**Affiliations:** 1 Department of Neurology, Victor Babes University of Medicine and Pharmacy Timisoara, Eftimie Murgu Sq. No. 2, 300041 Timișoara, Romania; mihaelasimu6713@gmail.com; 2 Department of Neurology, Clinical Emergency County Hospital Timisoara, Bd. Iosif Bulbuca No. 10, 300736 Timisoara, Romania

**Keywords:** Parkinson’s disease, cognition, screening, Parkinson’s disease-cognitive rating scale, systematic review

## Abstract

The aim of the present systematic review was to examine the evidence on the accuracy and psychometric properties of the Parkinson’s Disease-Cognitive Rating Scale (PD-CRS) for evaluating the presence of cognitive impairment in patients with Parkinson’s disease (PD) as well as to highlight the quality and quantity of research available on the use of the PD-CRS in this population. We searched four databases from inception until July 2020. Eight studies, published between 2008 and 2020, met the inclusion criteria: One cross-sectional study in which participants were assessed with the index test (PD-CRS) and a reference standard diagnostic assessment, in accordance with the Level II criteria of the International Parkinson and Movement Disorder Society (MDS); one case-control study comparing the PD-CRS to an extensive battery of tests (i.e., MDS Level II diagnosis); and six studies comparing the PD-CRS to other short cognitive batteries. In patients with Parkinson’s disease, the PD-CRS test provides information about cortical and sub-cortical cognitive functions. Even if it demonstrated good psychometric properties, the results regarding the optimal threshold for detecting mild cognitive impairment and dementia in PD are somewhat inconsistent. Further cross-sectional studies are necessary to examine the optimum cut-off score for detecting cognitive dysfunction in PD patients.

## 1. Introduction

Neurological disorders are a leading global cause of disability. A recent systematic review revealed that, among neurological disorders, Parkinson’s disease (PD) was the fastest growing in prevalence, disability, and deaths [1]. For example, in 2016, the overall number of individuals with PD globally was 2.4 times higher than in 1990. Worldwide, it has been estimated that 6.1 million people had PD; the disease caused 211,296. deaths, and 3.2 million disability-adjusted life-years (DALYs). In addition, as the population ages and life expectancy increases, it is expected that the number of PD cases will double in the coming generation [1].

Cognitive impairments are frequent in patients with PD, even early in the disease. A significant number of individuals eventually develop cognitive impairment, most prevalent being mild cognitive impairment (PD-MCI)—the intermediate stage between normal cognition and dementia—Ranging from 15% to 53% across studies [2]. Furthermore, it has been estimated that 20–42% of patients present PD-MCI at the time of PD diagnosis [3,4].

PD-MCI is associated with the development of dementia (PD-D) [5,6]. However, reports from longitudinal studies are heterogenous. Some patients with PD-MCI may revert to normal cognition during follow up, ranging from 11% [7] to 28% [8]. Nonetheless, after 5-years of follow-up, 39% to 50% of patients with PD-MCI develop dementia [7,8,9].

Community-based studies have reported that the incidence of PD-D ranges from 95.3 to 112.5 per 1000 patient-years [10,11,12], and approximately 10% of PD patients develop dementia per year. Compared to non-PD subjects, the relative risk for developing dementia in PD ranges from 1.7 to 5.9 [11,12,13], depending on variables such as patient selection procedures and the use of different risk estimation methods. A study investigating the incidence of dementia among patients with PD, found that the odds of being diagnosed with dementia was 3.5 (95% CI = 1.3–9.3) [14].

Furthermore, recent studies have reported that cognitive deficits may be detected even before the onset of motor symptoms (i.e., in the prodromal phase of PD), poor global cognition being associated with an increased risk of developing PD [15]. Cognitive impairments have also been reported in individuals at risk of developing PD with hyposmia or rapid eye movement (REM) sleep disorders [16], as well as in unaffected relatives of PD patients who had hyposmia and abnormal DaT scans [17]. All these findings question the definition of PD-D, which classically is considered to develop after the onset of motor symptoms.

Cognitive impairment in PD includes a spectrum of severity, ranging from mild cognitive difficulties—which may present early in the disease course—to severe dementia, most frequently occurring after several years following the onset of motor symptoms [18]. The onset of cognitive dysfunction is insidious, with a slowly progressive course.

In PD patients, all cognitive domains may be affected, but greater impairment is typically found in executive functions, attention, memory, as well as visuospatial skills, with relatively preserved core language functions. Overall, the most frequent subtype of PD-MCI is non-amnestic, single-domain impairment [6]. In PD-D, most patients present a dysexecutive cognitive profile, which is often associated with behavioral features like mood changes, apathy, hallucinations, and delusions [19].

Among the executive functions, including working memory, attention, and inhibitory control [20,21,22,23], response inhibition presents distinct importance, as it is associated with a broad clinical picture, including cognitive and motor symptoms. Deficits in inhibitory control impact the patient’s ability to pursue future-oriented goals, with a negative impact on the quality of life [20], and it has been found to be a sensitive outcome measure for diagnosis and progression of PD [23,24].

The defining feature of PD-MCI and PD-D is that cognitive impairment develops in the context of established PD. Therefore, based on an empirical approach, the first step in diagnosis is establishing the diagnosis of idiopathic PD. The PD-MCI may be present at the time of PD diagnosis, but the PD-D should be diagnosed when dementia develops within the context of established PD. The diagnosis of dementia with Lewy Bodies (DLB) is recommended when dementia precedes or coincides within 1 year of the apparition of motor symptoms [19].

In recent years, the International Parkinson and Movement Disorder Society (MDS) has created a task force that developed a unified definition of PD-MCI, based on expert consensus [6]. PD-MCI was defined as an insidious decline in cognitive functions reported by the patient or caregiver or noted by the clinician, not caused by other comorbidities. Contrary to dementia, the cognitive impairments do not interfere with the functional independence of the patient [6].

In addition, the MDS adopted a two-level definition of PD-MCI. For a level I assessment, PD-MCI is diagnosed based on an abbreviated cognitive evaluation, either with a global scale such as the Parkinson’s Disease-Cognitive Rating Scale (PD-CRS) or a limited range of neuropsychological tests (e.g., including only one test per cognitive domain or those evaluating fewer than five cognitive domains). If a limited number of tests are used, the impairment must be documented in at least two tests [6].

The level II definition is based on an extensive assessment of each of the five cognitive domains (i.e., attention, working memory, executive functions, memory, visuospatial skills and language). PD-MCI is diagnosed if the patients present deficits in at least two tests within or across the five cognitive domains [6].

The MDS has also developed guidelines for diagnosing PD-D [19,25,26]. The clinical criteria for PD-D include four aspects: “Core features, associated clinical features, features that make the diagnosis uncertain, and features that are not compatible with the diagnosis of PD-D”. If a patient presents core and associated features, but they have no features that make the diagnosis uncertain or features that suggest other conditions as a cause of cognitive impairment, they are diagnosed with probable PD-D. If a patient presents core features, without features that are not compatible with the diagnosis of PD-D (i.e., vascular dementia), but clinical features are atypical, or uncertainty factors exist, possible PD-D is assigned [19,25,26].

Similar to PD-MCI, there are two levels of application. First, the diagnosis of PD-D can be established with a bedside cognitive screening test (level I). The second approach (level II) is a more detailed method that assesses four domains: Global cognitive functions, subcortico-frontal features, instrumental (cortically mediated) functions, and neuropsychiatric symptoms [19,25,26].

Recently, the MDS invited an international group of experts to review and critique the scales evaluating global cognitive functions in PD [18]. A scale was “recommended” if it was studied in PD, if there were data on its use beyond the developers, and if it was reported to be reliable, valid, and sensitive to cognitive changes in PD. Three scales met all of these criteria: The Montreal Cognitive Assessment (MoCA) test [27], the Mattis Dementia Rating Scale Second Edition (DRS-2), [28], and the PD-CRS [29]. Two scales were “recommended with caveats”, as their properties were found to be adequate but some of the measurement characteristics were not investigated: Mini-Mental Parkinson (MMP) [30] and Scales for Outcomes in Parkinson’s Disease-Cognition (SCOPA-COG) [31]. Six scales were only “suggested”: Alzheimer’s Disease Assessment Scale–Cognition (ADAS-COG), Cambridge Cognitive Assessment-Revised (CAMCOG-R), Mini-Mental State Examination (MMSE), Parkinson Neuropsychiatric Dementia Assessment (PANDA), Parkinson’s Disease Dementia-Short Screen (PD-SS), and Repeatable Battery for the Assessment of Neuropsychological Status (RBANS).

All these scales may be available in primary healthcare settings, although some are protected by copyright (i.e., ADAS-COG, CAMCOG-R, DRS-2, MMSE, PANDA, and RBANS). Nonetheless, they may only be used for the level I diagnosis of cognitive impairment, while level II diagnosis requires a more extensive cognitive assessment. However, comprehensive neuropsychological testing is not widely available, requires highly trained personnel, and is time consuming. Hence, brief screening tests that are sensitive, easily accessible, and may be administered by healthcare providers across a range of settings would be useful and may acceptably cover multiple cognitive domains [18]. Therefore, a global cognitive scale is sufficient for the level I diagnosis of PD-MCI and PD-D.

The MoCA test has been reported to present high sensitivity and specificity for detecting mild cognitive impairment (MCI) in the older adult population [27]. It is a short bedside test, its administration time being approximatively 10 min. The MoCA evaluates executive functions, memory and attention, visuospatial functions, naming, language, abstraction, and orientation. The maximum score is 30 points, and a cut-off of 25 or lower indicates cognitive impairment. At present, this threshold is widely used for detecting cognitive impairment. However, recent systematic reviews investigating its use in patients with different types of cognitive disorders have revealed that lowering the threshold offers a better balance between true positive and false positive results [32,33,34,35,36]. In order to minimize practice effects, alternative versions of the MoCA have been developed. Furthermore, it has been translated into multiple languages. The MoCA demonstrated excellent internal consistency in patients with MCI, with a Cronbach’s alpha of 0.83 [27]. It has presented a good test-retest reliability, with a mean change in MoCA scores between two evaluations of 0.9 points [27]. Online training and certification can be obtained on the MoCA website (https://www.mocatest.org/).

The DRS-2 scale [28] investigates attention, initiation and perseveration, visuospatial skills, conceptualization, and memory. The administration time is approximately 15–30 min, and total scores are adjusted for age and education level. The test is copyrighted and available from Psychological Assessment Resources (https://www.parinc.com/Products/Pkey/90). It has good internal consistency (Cronbach’s alpha of 0.82) and test-retest reliability (0.97) [37]. The scale demonstrated to be a valid instrument for distinguishing patients with Alzheimer’s disease (AD) and PD-D from those with MCI or healthy controls [38]. The scale presents good clinimetric properties, and normative data and cut-offs are available for PD-D and PD-MCI. Nonetheless, it also presents some limitations: It has potential ceiling effects, normative data for individuals with low education are not available, it does not have alternate versions, and it has increased costs in comparison with free-to-use scales [18].

The PD-CRS was specifically developed to provide comprehensive cognitive screening for PD patients. It evaluates both cortical and subcortical functions, including naming and copy drawing of a clock, verbal memory, attention, working memory, visuo-spatial functions, alternating, and action fluency [29]. Furthermore, there are available translations into different languages (English, Italian, Spanish, Dutch, and Chinese), and an alternate version has also been published [18]. Furthermore, population-based norms are available for the Italian population [39]. Its administration time is approximatively 20 min. The PD-CRS is in the public domain (https://www.movementscales.com/), and training is also available. The MDS recommends it “for all types of studies, including screening, prevalence, correlation studies, and treatment trials” [18].

The diagnosis of cognitive impairment in PD also necessitates neuroimaging. Although patients with PD-MCI and PD-D present a similar pattern of brain atrophy, in magnetic resonance imaging (MRI), it is distinct in PD patients with normal cognition, with hippocampal, prefrontal, occipital, and parietal brain atrophy [40]. Furthermore, an Alzheimer’s disease-like pattern of brain atrophy has also been reported in patients with PD-MCI and PD-D [41]. Therefore, cognitive impairment from PD might also be due to the development of associated AD pathology. In addition, MRI is used for the differential diagnosis with other diseases (such as vascular dementia).

Although the National Institute on Aging-Alzheimer’s Association workgroups recommend the inclusion of biomarkers for the diagnosis of other types of cognitive impairments such as Alzheimer’s disease [42], there is no reliable diagnostic neural biomarker in PD [43].

Correctly identifying cognitive impairment in PD patients enables adequate interventions. A recent MDS systematic review concluded that, in PD-MCI, the monoamine oxidase B (MAO-B) inhibitors rasagiline and rivastigmine presented an acceptable risk of adverse events, without specialized monitoring, but there is still insufficient evidence on their efficacy [44]. Non-pharmacological interventions, including transcranial direct-current stimulation (T-DCS) and cognitive rehabilitation are still under investigation [44,45].

In patients with PD-D, among acetylcholinesterase inhibitors, rivastigmine is considered clinically useful, while donepezil and galantamine are considered possibly useful [44]. In addition, another meta-analysis found that cholinesterase inhibitors slightly improved global impression and enhance cognitive function [46].

Nonetheless, despite the recent increasing research on effective therapies for cognitive impairment in PD, this area still lacks an adequate evidence base of high-quality studies.

In addition, anti-Parkinsonian medication may also affect cognition. A recent systematic review on the effects of currently prescribed anti-Parkinsonian treatments on cognition in patients with mild-moderate PD, who presented either normal cognition or PD-MCI, found that levodopa was beneficial on tests of attention, processing speed, working memory, executive functions, and episodic memory [47]. In contrast, Pramipexole was associated with a deterioration of episodic memory and impulse control, while selegiline was associated with a decline of global cognition over time and of concept formation. Rasagiline has been reported to provide some benefits for verbal fluency and working memory [47]. These findings are in line with the conclusions of an earlier narrative review on anti-Parkinsonian medication effects in mild PD [48]. Consequently, it is important to assess the cognitive status of the patients before initiation of anti-Parkinsonian therapy, as differentiating patients with cognitive impairment from patients without intact cognition provides a better understanding regarding the most favorable contexts to prescribe different types of medications.

A neuropsychological assessment has an important role in establishing baseline cognitive abilities, monitoring progression, and for the evaluation of the outcome of therapeutic interventions [6,49]. However, an extensive neuropsychological assessment necessitates considerable time and resource demands, and it is difficult to use as routine cognitive monitoring. Therefore, validated cognitive monitoring tools that can be easily incorporated into the clinical settings are needed.

PD-D is associated with a significant loss of functional independence, increased psychiatric comorbidity, worsened quality of life, and increased caregiver burden, earlier nursing home placement, and increased mortality [50,51]. Therefore, early detection of cognitive dysfunction and the identification of patients at risk of dementia are important. First, the use of adequate interventions might improve the patient’s status. Second, new disease-modifying treatments for PD and early interventions to slow or prevent PD-D may emerge. In addition, early diagnosis of cognitive impairment allows prognostic information that can help patients to plan their future better and enables healthcare providers to plan health and social needs for them [52].

The PD-CRS fulfills important feasibility criteria for use in clinical practice: It has a short administration time, is freely available and can be obtained along with training, has several translations, and an alternate version has recently been developed. In addition, it has been demonstrated to have good psychometric properties and evaluates a broad range of cognitive functions, being specifically designed for patients with PD.

Therefore, the PD-CRS may help identify patients with PD, which require further assessments and specific care. Nonetheless, false-positive test results may contribute to avoidable costs and potential harm resulting from the distress associated with unnecessary investigations. Consequently, it is important to determine the strength of the empirical evidence that supports the use of PD-CRS as a screening test for cognitive impairment in PD. In the present systematic review, we aim to examine evidence from different studies, in order to integrate the available information and provide data for rational decision making, highlighting possible answers, that are easily accessible to clinicians and healthcare providers. Furthermore, we aim to investigate the quality and quantity of evidence available about the PD-CRS in the PD population and to identify gaps in the evidence and ascertain where further research is needed.

## 2. Methods

We performed a systematic review following the Cochrane recommendations [53] and the guidelines of the Preferred Reporting Items for Systematic Reviews and Meta-Analysis (PRISMA) [54].

We searched MEDLINE/PubMed, Scopus, Latin American and Caribbean Health Sciences Literature (LILACS), and PsychINFO, from inception to July 2020. We performed an additional manual search on the PD-CRS website and by checking the reference lists of all relevant papers in order to identify possible additional studies.

We used the following keywords: “Parkinson’s Disease-Cognitive Rating Scale” OR the acronym “PD-CRS”, AND “Parkinson’s disease” (MeSH). These search terms were for PubMed, the primary source of citations. For other data sources, similarly structured search strategies were designed, as appropriate for each database. We did not apply search filters (collection of terms aimed at reducing the number of titles needed to be screened), as our goal was to generate an extensive list of studies that would be suitable for answering the research question. In addition, no language restriction was applied to our search.

Two authors independently assessed the title, abstract, and full text (when needed) of all retrieved citations and evaluated whether the study met the inclusion criteria. We resolved disagreements through discussion; participation of a third rater was not needed to address discordances.

In order to perform a systematic review of PD-CRS use in the context of PD, we selected all the studies where PD-CRS was used to assess the cognitive functions in patients with PD. The main types of eligible studies, using the PD-CRS were: (i) Cross-sectional studies in which participants received the index test (PD-CRS) and a reference standard diagnostic assessment, in accordance with the MDS Level II criteria; (ii) case-control studies comparing PD-CRS to a battery of tests (MDS Level II diagnosis); and (iii) studies comparing PD-CRS to other short cognitive batteries.

Currently, no in vivo reference standard is available for the diagnosis of PD-MCI or PD-D. Therefore, we included all studies recruiting adult (over 18 years old) participants that met the UK Brain Bank criteria for PD, or the MDS clinical diagnostic criteria for PD [55], in which the association between any full version of the PD-CRS score and cognitive impairment was assessed. The target condition was cognitive impairment, including PD-MCI [6] and PD-D [19,25,26].

We excluded studies with patients with other potential causes of cognitive impairment (e.g., AD, vascular dementia, central nervous system (CNS) tumor, trauma or infection, or metabolic abnormalities) or with secondary Parkinsonism (e.g., vascular, toxic, drug-induced, post-infectious, and so on) or atypical Parkinsonism (e.g., corticobasal degeneration, Lewy body dementia, progressive supranuclear palsy, or multiple system atrophy).

## 3. Results

**Figure 1:**
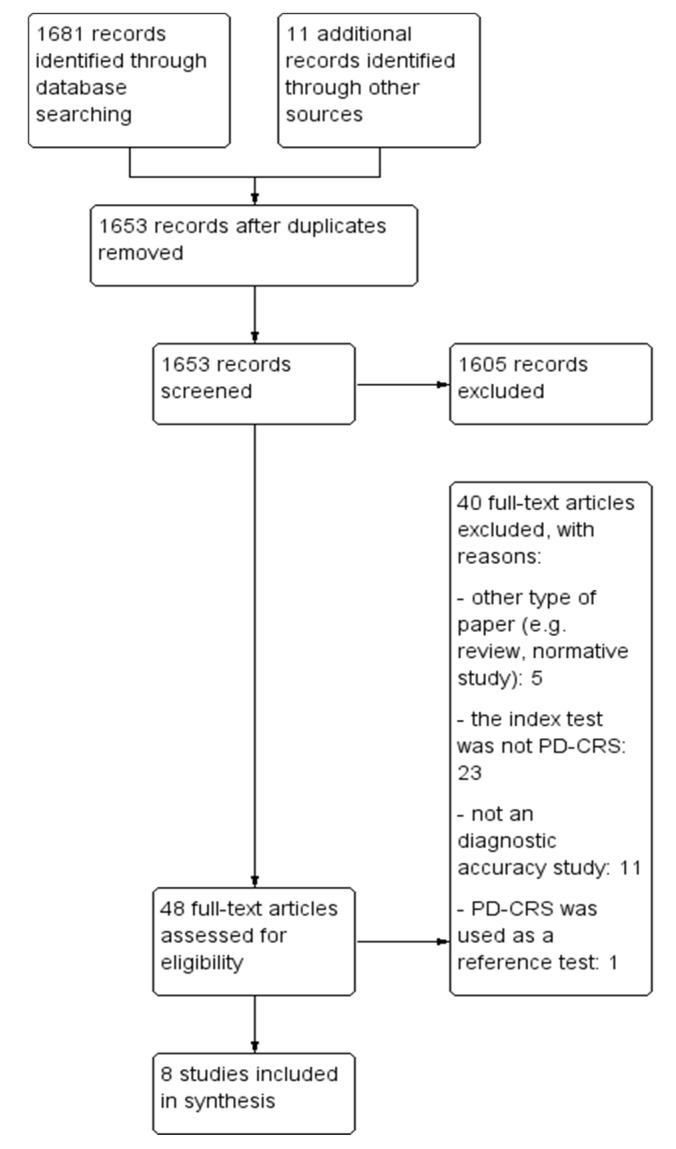
Study selection flow chart. PD-CRS: Parkinson’s Disease-Cognitive Rating Scale.

Our search resulted in 1692 records, from which we identified 48 unique that we assessed in full-text. We included in the present review eight studies: (i) One cross-sectional study, (ii) one case-control study comparing PD-CRS to a battery of tests, and (iii) six studies comparing PD-CRS to other short scales. The characteristics of the included studies are presented in Table 1. The PRISMA diagram detailing the selection process of studies is presented in Figure 1.

### 3.1. Methodological Quality of Included Studies

We assessed the methodological quality of the included studies. We considered the case-control studies [29,57,61] to be at high risk of bias, as in diagnostic accuracy studies, the use of a case-control design increases both the sensitivity and specificity of the index test [53].

With regard to the patient spectrum domain, the method of sampling patients for inclusion may cause important variations in diagnostic accuracy. The optimal study design should include a prospective, consecutive, or random series of patients that fulfill all selection criteria. The use of other sampling methods may lead to a high risk of introducing bias into the study [53]. Most of the studies did not report the method used to sample patients for inclusion [29,57,58,60,61,62]. A consecutive sample of PD patients was recruited in two studies [56,59]. Furthermore, three studies included only non-demented patients [58,60,61], and, therefore, the data obtained cannot be generalized. In terms of risk of bias, if, for example, a study does not include consecutive patients, more difficult cases could be excluded; this leads to an increased sensitivity and specificity of the index test, with a lower number of false positive or false negative test results [53].

Regarding the reference standard domain, some items of the PD-CRS were also present in the reference standard; consequently, incorporation bias was present, likely to increase the amount of agreement between index test and the reference standards results, overestimating the diagnostic accuracy of PD-CRS. This was especially evident in studies using the MoCA test as a reference standard, as some items such as clock drawing and verbal fluency are also included in the MoCA test [60].

The time period between the administration of PD-CRS and the reference standard was adequate in most studies [29,56,57,58,60,62], with a low risk of bias. Only two studies did not report the exact interval between tests [59,61]. Ideally, the index test and the reference standard were performed during the same study visit or in a short time interval. If there was a significant delay between tests, disease progression bias and recovery bias may occur. Patients could be misclassified due to benefit from treatment, progression of the disease, or occurrence of a new condition [53].

Four studies provided data on the “blinding” of the assessors, clearly reporting that it was a blind review [29,57,58,59]. Interpretation of the results of the PD-CRS may be affected by knowledge of the results of the reference standard (test review bias) and vice versa (diagnostic review bias). Both types of bias have been shown to increase the sensitivity of a test; however, no systematic effect on specificity has been observed [63]. Nonetheless, one paper reported that the PD-CRS test and the reference standard were undertaken in a specific order [62]; hence, the first test (PD-CRS) must have been interpreted blind to the results of the second (CDR). All the other studies were considered to present an unclear risk of bias [56,60,61].

The cognitive tests were administered by a neuropsychologist in most studies [29,56,57,58,60] and by a neurologist in one study [62].

Other potential limitations of the included studies are detailed in the Table S1 (Supplementary Materials).

### 3.2. Findings

The present review included eight studies, published between 2008 and 2020. The studies were performed in five different countries. Samples ranged in size (from 46 to 235 participants), gender (54.66% males to 71.74% males), median age, educational level, PD-CRS scores, disease duration and severity, and medication received. The characteristics of the included studies are presented in Table 1.

In the selected studies, the Cronbach’s alpha of PD-CRS was reported to range from 0.82 to 0.85. For detecting PD-D, although the initial validation study recommended the use of a threshold of ≤ 64 [29], other studies found that the optimal cut-off was ≤ 62 [59] or ≤ 73.5 [62]. When used to detect PD-MCI, cut-offs of ≤ 81 [57,58], 80.5 [62], or ≤ 101 [61] have been reported to be useful for optimal screening.

#### 3.2.1. Cross-Sectional Studies in Which Participants Received the Index Test (PD-CRS) and a Reference Standard Diagnostic Assessment, in Accordance with the MDS Level II Criteria (Table 1 and Table S1)

To date, only one cross-sectional study assessed the validity of PD-CRS as a screening tool for cognitive dysfunction in PD, using a comprehensive neuropsychological assessment as a reference standard.

The study of Fernandez-Bobadilla et al., [58] evaluated the clinical value of the original PD-CRS test and the alternative form of the scale (PD-CRS/AF) in detecting cognitive deficits in non-demented PD patients. The receiver operating characteristic (ROC) curve analysis indicated that the optimal threshold for PD-CRS was ≤ 81 (sensitivity 94%; specificity 73%) with an area under the curve (AUC) of 0.91. Similar results were obtained for PD-CRS/AF: The maximum cut-off accuracy for detecting PD-MCI was obtained with a total score 81, yielding to a sensitivity of 92%, a specificity of 73%, and an AUC of 0.887.

The authors found a strong correlation between the two versions of PD-CRS for the total score and separate sub-scores. There was no effect in relation to the order that participants completed the two tests, indicating that the two versions can be used in either order. Furthermore, the authors did not report any practice effects as a result of administering the two similar tests over a short period of time [58].

#### 3.2.2. Case-Control Studies Comparing PD-CRS to a Battery of Tests (MDS Level II Diagnosis); (Table 1 and Table S1)

We found only one case-control study assessed the validity of PD-CRS as a screening tool for cognitive dysfunction in PD, using a comprehensive neuropsychological assessment (MDS Level II criteria) as a reference standard.

The study of Koevoets et al., [61] evaluated the accuracy of the PD-CRS and the MDRS-2 for detecting PD-MCI in a sample of patients who were candidates for deep brain stimulation (DBS). Demographic influences were corrected with data from healthy controls. When assessed with an extensive neuropsychological battery, 27%of the patients presented with PD-MCI. The optimal cut-off for the PD-CRS was 101/102 (sensitivity = 88%; specificity = 64%; AUC = 0.83). The sensitivity and the specificity of the test were not improved by the demographical correction of scores. The AUC for demographically corrected scores was 0.80. At the optimal threshold of 101/102, sensitivity was 0.79 and specificity was 0.72 [61].

The results of the study indicated that PD-CRS was not superior to MDRS-2, even though the latter had a clear ceiling effect in subjects with normal cognition. However, the administration of PD-CRS might be preferred in clinical practice as its administration time is slightly shorter [61].

#### 3.2.3. Studies Comparing PD-CRS to Other Short Cognitive Batteries (Table 1 and Table S1)

We identified six studies directly comparing the PD-CRS with other short cognitive tests (MDRS, CDR, MMSE, and MoCA), the latter being used as a reference standard.

The initial validation study of PD-CRS [29] included a case-control design and was aimed to develop a comprehensive but practical cognitive assessment test for PD patients. In addition, the scale was designed to evaluate the fronto-subcortical and cortical cognitive functions, aiming to provide information that may assist in increasing the sensitivity and specificity in the diagnosis of PD-D, to separate subgroups of patients according to their pattern of cognitive impairment from the early stages of the disease, and to detect those patients with a higher risk of developing dementia [29].

The authors used the Clinical Dementia Rating Scale (CDR) as a reference standard. In addition, the subjects were assessed with the Mattis Dementia Rating Scale (MDRS) and a neuropsychological battery with validated cognitive tests, evaluating the same cognitive functions as those assessed by the PD-CRS [29].

The total scores of the final version of the PD-CRS presented a strong concurrent validity with the total scores of the MDRS, with an intraclass correlation coefficient (ICC) of 0.87 (CI 95%: 0.82–0.90). The individual items, total, cortical and subcortical scores of the PD-CRS also demonstrated a high test-retest and inter-rater reliability, the ICC ranging from 0.75 to 0.94. In addition, the test was found to present a high internal consistency (Cronbach’s alpha = 0.82) [29].

The items of the scale presented no floor effects, indicating that patients obtained a minimum score only after severe cognitive impairment was reached. When the authors analyzed the PD group as a whole, only the “cortical-type” items presented a ceiling effect. This effect disappeared when the PD-D group was analyzed separately [29].

The optimal cut-off score for the screening of PD-D was ≤ 64, yielded high sensitivity (94%) and specificity (94%), as well as good positive and negative predictive values (PPV = 0.91, NPV = 0.96). The AUC was 0.98 (CI 95%: 0.96–0.99). The ROC curve analysis discriminating PD-MCI from patients with intact cognition revealed a moderate sensitivity and specificity for total PD-CRS scores (sensitivity = 0.73, specificity = 0.84) or subcortical PD-CRS scores (sensitivity = 77%, specificity = 71%) [29].

The overall administration time of the PD-CRS was 16 ± 3.6 min in the PD group with intact cognition and 24 ± 7.8 min in the PD-D group [29].

In conclusion, the initial study on PD-CRS found that the scale was a valid, reliable, and useful neuropsychological test that accurately diagnoses PD-D; in addition, it was able to detect mild fronto-subcortical dysfunctions in non-demented patients. Furthermore, the authors showed that the transition from PD-MCI to PD-D was characterized by the addition of “cortical type” cognitive defects upon a progressive and predominant fronto-subcortical impairment [29].

A separate validation study of PD-CRS was performed by Martinez-Martin et al. [56]. The cross-sectional observational study compared the PD-CRS with MMSE, Scopa-Cog, and the Impression of Severity Index for Parkinson’s Disease (CISI-PD) scales. Nonetheless, the authors did not use any formal criteria for PD-MCI or PD-D (e.g., MDS criteria or MCI criteria).

The authors reported a good Cronbach’s alpha (0.85). A significant ceiling effect was noted in the PD-D group for the cortical items. However, no ceiling or floor effect was found for the subcortical items or the total scores of PD-CRS. The total PD-CRS score presented a high correlation with the Scopa-Cog scores but was less corelated with the MMSE scores. In addition, the PD-CRS scores were significantly lower in patients with increased severity of cognitive symptoms as evaluated by CISI-PD [56].

The case-control study of Fernandez Bobadilla et al., [57] investigated the sensitivity to longitudinal change of the PD-CRS in non-demented PD patients and provided a cut-off value of the scale for differentiating the patients with normal cognition and PD-MCI patients. The authors categorized the global cognitive status of the subjects using the Mattis Dementia Rating Scale-2 (MDRS-2), the CDR scale, and the Cognitive Impairment item in part I of the MDS-UPDRS (MDS-UPDRS cog-I).

The AUC analysis (AUC = 0.85; 95% CI: 0.80–0.90) revealed that the optimal cut-off score was 81 (sensitivity = 79%; specificity = 80%; PPV = 0.59; NPV = 0.91) and a range of change from 10 to 13 points on the PD-CRS total score was suggestive of clinically significant change. In patients with PD-MCI, clinical worsening was indicated by a decrease of 14 points, while an increase of 11 points was the minimum change for a relevant improvement in the clinical status of a patient [57].

The best variables to independently differentiate PD-MCI from patients with PD and intact cognition were found to be the PD-CRS total score and age. Other factors, including education, PD evolution, H&Y staging, UPDRS-III scores, and depression scores, were not predictive of PD-MCI [57].

The main objective of the cross-sectional study of Samat et al., [60] was to investigate the prevalence of PD-MCI among 46 PD patients using the PDCRS and MoCA test, the Level I MDS criteria [6], and to correlate the presence of cognitive dysfunction with measurable biomarkers such as ApoE4 and plasma a-synuclein.

In this study, when assessed with the MoCA test, 26 (56.5%) of the PD patients were diagnosed with PD-MCI, while 20 (43.5%) of patients had intact cognitive functions.

Based on the PD-CRS, 39.2% of the PD patients presented normal cognition, 36.9% were in the PD-MCI category, and 23.9% had been diagnosed as PD-D. This was due to the fact that 11 out of 26 patients (42.3%) with MCI in MoCA were re-categorized as PD-D using the PD-CRS. In addition, 5 (25%) out of 20 patients with intact cognition in MoCA were reclassified as PD-MCI using the PD-CRS. However, 3 of 26 patients with PD-MCI in MoCA were classified as having intact cognition by the PD-CRS, giving a false positive value for MoCA of 11.5%. The bivariate analysis revealed a positive and significant correlation between the MoCA and PD-CRS scores [60].

The authors concluded that, in comparison to MoCA, the PD-CRS might be a better tool to detect PD-MCI, probably due to its ability to evaluate not only fronto-subcortical but also posterior cortical impairments [60].

The cross-sectional study performed by Serrano Duenas et al. [59] investigated the properties of PD-CRS using the MMSE as a reference standard, in accordance with the MDS Levell I criteria (Serrano Duenas et al., 2016) in diagnosing PD-D. The internal consistency of PD-CRS was found to be good, with a Guttmann’s *λ* value of 0.821. Four of the items, including sustained attention, working memory, immediate verbal memory, and alternating verbal fluency presented a floor effect, while one item presented a ceiling effect (clock copying). The scale adequately differentiated patients with and without dementia (Kruskal-Wallis; *p* ≤ 0.000). The AUC was 0.899; using a cut-off score of 62, the scale presented a sensitivity of 94%, and a specificity of 99%, with a PPV of 96.7%, an NPV of 98.1%. The positive likelihood ratio was 94, and the negative likelihood ratio was 0.06 [59].

The cross-sectional study of Tan et al. [62] investigated the reliability and validity of the Chinese version of PD-CRS, as well as the optimal cut-off scores for diagnosing PD-MCI and PD-D. As a reference standard, the authors used the CDR scale.

The PD-CRS presented high internal consistency (Cronbach’s Alpha = 0.840). The intraclass correlation coefficient (ICC) of test-retest reliability was 0.906 and the ICC of inter-rater reliability was 0.899.

The patient’s cognitive abilities were also assessed with MDRS. The PD-CRS presented a fair concurrent validity with MDRS, with an ICC of 0.731 [62].

All fronto-subcortical items presented significant decreases in PD-MCI compared with the PD group with intact cognition, but the cortical items (e.g., confrontation naming and clock copying) did not. In the PD-D group, all of the frontal-subcortical and instrumental-cortical functions presented a significant decline compared with the PD patients with normal cognitive functions [62].

The optimal cut-off score for diagnosing PD-MCI was 80.5, with a sensitivity of 75.7% and a specificity of 75.0%. For a diagnosis of PD-D, the optimal threshold was 73.5, with a sensitivity of 89.2% and a specificity of 98.9% [62].

In addition, the authors reported the presence of nonfloor effects for the total, subcortical, and cortical scores of the PD-CRS when analyzed in all PD patients, specifically PD with intact cognition and the PD-MCI subgroup. In the PD-D subgroup, a floor effect was found in items of immediate and delayed free-recall verbal memory, confrontation naming, sustained attention, working memory, and alternating verbal fluencies, indicating that severe impairment in those cognitive functions was common in PD-D patients. When analyzing the whole sample of PD patients, the authors found a ceiling effect in confrontation naming (15.2%), clock drawing (32.6%), and copying a clock (72.8%). In the PD group with normal cognition, the ceiling effect was present in the items confrontation naming (21.6%), sustained attention (21.6%), clock drawing (54.1%), and copying a clock (86.5%). In the PD-MCI group, a ceiling effect was demonstrated only for the clock copying item (20.5%) [62].

For discriminative validity, significant differences were noted in total PD-CRS, frontal-subcortical functions, and instrumental-cortical functions, and each PD-CRS item scores among PD patients with intact cognition, PD-MCI, and PD-D groups [62].

## 4. Discussion

The present systematic review enabled us to make several key observations.

In the selected studies, the authors found PD-CRS to have high discriminative power for differentiating the PD patients with cognitive impairment from cognitively intact PD patients [56,58,59,60,62], or from healthy controls [29,57,61].

The PD-CRS was found to present a good internal consistency, with a Cronbach’s alpha greater than 0.82 in multiple studies [29,56,64], and a good test-retest and interrater reliability, ranging from 0.75 to 0.94 [29,59,62]. The scale was reported to have a good correlation with other short cognitive assessment instruments such as the MMSE [56,59], Scopa-Cog [56], DRS-2 [57], MoCA [60], and CISI-PD [56]. In addition, PD-CRS was shown to be correlated with age and education [59,61], with PD-CRS scores being significantly lower in older patients, with low education levels [56].

The scale has been demonstrated to be sensitive to change, with a decrease from 10 to 13 points in the total score, indicating a clinically significant change in cognitive abilities [57].

The optimal cut-off for detecting PD-MCI has been reported to be 81 [57,58], 80.5 [62], or ≤ 101 [61]. Similarly, for detecting PD-D, several cut-offs were reported to be optimal: ≤ 64 [29], ≤ 62 [59], or ≤ 73.5 [62].

These differences could be due to the heterogeneity of the PD samples and the use of different reference standards. Even if the authors compared the PD-CRS with an extensive neuropsychological battery using the MDS Level II criteria, this could have induced some bias. The MDS recommends the diagnosis of cognitive impairment if a patient presents a performance between 1 to 2 SDs below age, education, gender, and culturally corrected norms. However, it has been demonstrated that differences in the cut-off scores utilized can lead to a wide range of prevalence estimates for PD-MCI [65]. It has been reported that the best practice to minimize the rate of false positive results was to require deficits of at least −1.5 SD in two scores within any single domain (resulting in 30% PD-MCI) or deficits of at least −1.5 SD in two scores from different domains (37% PD-MCI). Furthermore, studies have demonstrated that, assuming a normal distribution of test scores, 7% of individuals scoring 1.5 SD or more SD below the mean would be falsely diagnosed to have cognitive impairment (i.e., false positives). The requirement for impaired performance on at least two tests diminishes the risk for false positives, but not false negatives [6,65]. In addition, some errors are caused by two frequent methods to increase the sensitivity of a test regarding mild cognitive impairments: For example, extensive neuropsychological batteries have higher false positive rates than individual tests, as they include multiple comparisons. The likelihood of an abnormal score increases as the number of tests performed per cognitive domain and the number of assessed domains increases (i.e., diagnosing an individual with intact cognition as impaired). In addition, the use of high cut-off scores (i.e., z-scores with a threshold of 1 SD) increase the overlap between critical portions of test score distributions in subjects with and without disease [66,67]. Increased sensitivity is inevitably based on a reduction in specificity. Consequently, false-positive cases can bias the prevalence estimates and will decrease the power of analytical estimates [65].

Although the PD-CRS has been designed specifically for PD patients, it also presents some possible limitations. First, the scale examines only some aspects of executive functions, but it has no items for inhibitory control. The patients with PD might present inhibitory deficits on executive control tasks necessitating inhibition of habitual or prepotent responses for selecting the proper responses, such as on the stop-signal task, Stroop, Go/No-Go, or random number generation tests [21,24]. Nonetheless, tests for inhibitory control are not available for paper and pencil tests and it would be difficult to incorporate them into routine cognitive testing. Second, the PD-CRS evaluates the executive functions only with performance-based measures; it has been demonstrated that performance-based and rating measures of executive function (questionnaire-based measures) evaluate different clinical aspects of executive function. Therefore, these measures cannot be used interchangeably as assessment tools of executive functions [68,69].

To date, research on the use of the PD-CRS in patients with PD is somewhat limited. There has only been one cross-sectional study [58], and one case-control study [61], assessing the accuracy of PD-CRS in screening for cognitive impairment in this population using an extensive neuropsychologic examination as a reference standard, in accordance with the MDS Level II criteria. The rest of the studies investigated the PD-CRS test using only a short cognitive battery as a reference standard. Furthermore, among the short scales used as reference standards, MoCA and CDR-2 are “recommended”; while the Scopa-Cog has been “recommended with caveats,” and the MMSE is only “suggested” by the MDS [18]. Therefore, the results must be interpreted with caution. In addition, data on the optimal cut-off for detecting PD-MCI or PD-D are inconsistent.

Although the PD-CRS is “recommended” by the MDS as “useful for all types of studies, including screening, prevalence, correlation studies, and treatment trials” [18], the research regarding its use is quite limited, and any cognitive assessment with this brief test should require additional, comprehensive testing, in order to determine the severity of cognitive dysfunction.

The present review endorses the main potential benefit of PD-CRS, as a test that has shown promise in significantly decreasing the cognitive assessment time and costs, with good preliminary psychometric properties. However, the optimal cut-off should be further investigated. Furthermore, different thresholds should be tested in patients with different cultural and educational backgrounds, and those speaking different languages. In addition, it is necessary to consider the value of PD-CRS in a diagnostic workup such that clinicians may understand how to use this screening test to obtain relevant outcomes for patients, such as the benefits of earlier diagnostics. Nonetheless, an abnormal screening result should prompt for further assessment with a full neuropsychological assessment, until additional data are provided regarding the optimal cut-off.

Our study has certain limitations. A meta-analysis was not performed because the literature search revealed a low number of studies, with significant heterogeneity among these studies, regarding patient samples, demographic differences, language, and cultural and educational background.

## 5. Conclusions

Despite the limitations mentioned above, our study represents the first systematic review of the literature published in this field and outlines an accurate state of knowledge on the use of the PD-CRS in patients with PD. The scale deserves closer scrutiny, in order to assess its properties in individuals with PD, and further studies are required to determine whether the PD-CRS fills the needed role of assessing the cognitive status in PD. In addition, further cross-sectional studies are necessary in order to examine and determine the optimum cut-off score for detecting the presence of cognitive dysfunction.

## Figures and Tables

**Table 1:** Characteristics of included studies.

Study	Country of Origin	Study Type	Sample of PD Patients	Gender Men (%)	Age	Education	PD-CRS	UPDRS III	Hoehn & Yahr	L-Dopa mg/day	Total LED	Disease Duration	Other Scale
Pagonabarraga 2008 [29]	Spain	Case-control Comparison with another scale (CDR, MDRS)	92	59.3	71.2 ± 9.1	8.9 ± 5.3	8.2 ± 5	III 25.6 ± 12	1–8.8% 2–38.5% 3–39.6% 4–13.1% 5–0%	609.7 ± 408	774.7 ± 460	8.2 ± 5	MDRS 123 ± 17
Martinez-Martin 2009 [56]	Spain	Cross-sectional Comparison with other scale (MMSE, Scopa-Cog)	50	66	63.6 ± 9.3	9 ± 5.7	88.7 ± 19.8		1–12% 2–58% 3–20% 4–10% 5–0%	−	−		Scopa-Cog 24.3 ± 6.5 MMSE: 28.0 ± 2.9
Fernandez-Bobadilla 2013 [57]	Spain	Case-control Comparison with other scale	235 (145 NC, 89 PD-MCI)	57.3	67.60 ± 9.1	9.69 ± 5.1	83.26 ± 14.7	19.90 ± 9.7	1–25.2% 2–64.1% 3–9.8% 4–0.9% 5–0%	417.91 ± 398.5	596.91 ± 473.3	5.25 ± 4.6	MDRS-2 134.96 ± 7.7
Fernandez-Bobadilla 2017 [58]	Spain	Cross-sectional Comparison with a neuropsychological battery	75 (50 NC; 25 PD-MCI)	54.66	68.2 ± 10	NC: 13.1 ± 1.6 PD-MCI: 9.0 ± 4.1	88.61 ± 15.83	NC: 16.3 ± 11.0 PD-MCI: 24.7 ± 8.0	NC: 1.9 ± 0.4 PD-MCI: 2.1 ± 0.4	−	−	−	−
Serrano-Duenas 2016 [59]	Ecuador	Comparison with other scale Cross-sectional	223	69.9	69.4 ± 9.9	9.2 ± 4.7	76.0 ± 19.3	−	1–4.93% 2–21.9% 3–62.33% 4–10.76% 5–0%	716.9 ± 306.7	−	7.7 ± 5.4	MMSE 23.4 ± 4.9
Samat 2017 [60]	Malaysia	Comparison with other scale (MoCA) Cross-sectional	46 (20 NC, 26 PD-MCI	63	NC: 64 (58–65) PD-MCI: 63 (58–69)	Secondary level: NC: 12 (41.4%) PD-MCI: 17 (58.6%)	NC: 90 ± 11 PD-MCI: 68 ± 12	−	1–32.6% 2–56.5% 3–10.9% 4–0% 5–0%	−	NC: 225 (115.6–300.0) PD-MCI: 409.5 (200.0–707.8)	4 (3–7)	MoCA: NC: 44% PD-MCI: 56%
Koevoets 2018 [61]	Netherlands	Case control Comparison with a neuropsychological battery	125 (91 NC; 34 PD-MCI)	62.4	62.3 ± 6.9	(ISCED) education level of 4 (mean = 4.2, SD = 1.1)	97.4 ± 15.4	19.9 ± 9.0	−	−	−	−	MDRS-2 139.4 ± 4.6
Tan 2020 [62]	China	Cross-sectional Comparison other scale (CDR)	93 (37 NC, 44 PD-MCI, 11 PD-D)	71.74	NC: 68.08 ± 6.2 PD-MCI: 69.82 ± 6.366 PD-D: 71.27 ± 4.563	NC: 12.35 ± 2.879 PD-MCI: 11.63 ± 3.441 PD-D: 10.73 ± 2.284	75.32 ± 17.818	NC: 12.89 ± 8.906 PD-MCI: 20.48 ± 13.473 PD-D: 26.00 ± 11.773	−	−	NC: 323.97 ± 249.571 PD-MCI: 430.73 ± 287.325 PD-D: 540.91 ± 301.719	NC: 5.32 ± 5.716 PD-MCI: 5.18 ± 3.598 PD-D: 7.82 ± 3.401	MDRS NC: 138.16 ± 6.265 PD-MCI: 131.43 ± 9.260 PD-D: 114.27 ± 15.755

PD: Parkinson’s disease; PD-CRS: Parkinson’s Disease-Cognitive Rating Scale; UPDRS: Unified Parkinson’s disease rating scale; LED: levodopa equivalent dose; CDR: Clinical Dementia Rating Scale; MDRS: Mattis Dementia Rating Scale; MMSE: Mini-Mental State Examination; MoCA: Montreal Cognitive Assessment; Scopa-Cog: Scales for Outcomes in Parkinson’s Disease-Cognition; MDRS-2: Mattis Dementia Rating Scale Second Edition; NC: normal cognition; PD-MCI: Parkinson’s disease mild cognitive impairment; PD-D: Parkinson’s disease dementia.

## Supplementary Materials

The following are available online at https://www.mdpi.com/2076-3425/10/9/588/s1, Table S1: Summary of studies using PD-CRS as an instrument for the cognitive assessment of participants with Parkinson’s disease.
