# Supplementary material for: Parkinson’s Disease-Cognitive Rating Scale for Evaluating Cognitive Impairment in Parkinson’s Disease: A Systematic Review
Source: Brain Sci. 2020 Aug 25;10(9):588. doi: 10.3390/brainsci10090588 (PMC7565957; doi:10.3390/brainsci10090588)
Supplement: Supplementary file 1 [file brainsci-10-00588-s001.pdf]

Supplementary

Table S1. Summary of included studies.

Study	Objective	Methods	Results and conclusions considering PD-CRS	Limitations
Pagonabarraga 2008 [29]	To develop the PD-CRS, a new PD-specific cognitive scale aiming to capture the whole spectrum of cognitive functions impaired over the course of PD.	The study included 92 patients with idiopathic PD that were prospectively recruited from a sample of outpatients regularly attending a Movement Disorders Clinic.<br>Patients were classified as cognitively intact (CgInt), PD-MCI or PDD.<br>Intact cognition was diagnosed when patients had a score of 0 on the Clinical Dementia Rating Scale (CDR), PD-MCI when the score was 0.5, and PDD when the score was 1 and when they met criteria for PDD on the Diagnostic and Statistical Manual of Mental Disorders, revised Fourth Edition (DSM IV-TR).<br>A neurologist administered the Mattis Dementia Rating Scale (MDRS) and classified patients into cognitive groups according to the CDR.<br>A neuropsychologist blinded to the MDRS and CDR scores administered a comprehensive neuropsychological battery with validated cognitive tasks that assessed the same cognitive domains as those evaluated by the PD-CRS.<br>The control group consisted of 61 age-, sex- and education-matched healthy subjects, most of whom were spouses or	• The PD-CRS showed a strong concurrent validity with the MDRS, a test of global cognitive function that is specifically useful in PD.<br>• The study population consisted of 30 patients with intact cognition, 30 patients with PD-MCI and 32 patients with PDD.<br>• One-way ANOVA showed significant differences between PD groups for both age and education.<br>• Total scores of the final version of the PD-CRS showed a strong concurrent validity with the total MDRS scores (ICC = 0.87, 95% CI 0.82–0.90).<br>• The individual items, total, cortical and subcortical scores of the final version of the PD-CRS showed also a high test-retest and an inter-rater reliability, with ICC ranging from 0.75 to 0.94, as well as a high internal consistency (Cronbach's alpha 0.82).<br>• One-way ANOVA and Kruskal-Wallis test analysis showed significant differences between controls, patients with PD and intact cognition, PD-MCI, and	• The authors have not compared the PD-CRS with existing rating scales for cognitive dysfunction in PD. They cannot demonstrate that a scale with subcortical and cortical items performs better than a scale with subcortical items only.<br>• The absence of a consensus on the definition of PD-MCI (in 2008) determined the authors to adopt the MCI criteria used to classify subjects at risk for Alzheimer's disease and to categorize the subjects as CgInt or MCI subjects.<br>• It is unclear if a consecutive or random sample of patients was recruited

caregivers of the patients.	PDD groups for total ($p = 0.0002$;), cortical ($p = 0.0001$), and subcortical ($p = 0.0009$) PD-CRS scores.<br>• In the ANCOVA analysis, both total and subcortical PD-CRS scores did not separate controls from PD patients with intact cognition, but separated controls and CgInt from PD-MCI, and PD-MCI from PDD patients.<br>• PD-CRS cortical scores differentiated PDD from PD-MCI and CgInt, but did not differentiate PD-MCI from controls or CgInt patients. All these relationships had a significance level of $P < 0.01$.<br>• In the multivariate analysis, PDD were independently differentiated from the PD-ND group by the PD-CRS total score ($p = 0.0002$; OR = 0.79, 95% CI 0.70–0.89).<br>• For detecting PDD, the ROC curve showed that a cut-off score of 64 on the PD-CRS total score yielded a high sensitivity (0.94) and specificity (0.94), and positive and negative predictive values (PPV 0.91, NPV 0.96). The AUC was 0.98 (95% CI, 0.96–0.99).<br>• The ROC curve analysis to discriminate PD-MCI from CgInt patients yielded moderate sensitivity and specificity for total PD-CRS scores (sensitivity 0.73, specificity0. 84) or subcortical

			PD-CRS scores (sensitivity 0.77, specificity 0.71).	
Martinez-Martin 2009 [56]	To independently evaluate the psychometric properties of PD-CRS.	The present observational, cross-sectional study included 50 patients with PD. The subjects were assessed with Scales for Outcomes in Parkinson's Disease-Motor scale (SCOPA-Motor), Hoehn & Yahr (HY) staging, Clinical Impression of Severity Index for Parkinson's Disease (CISI-PD), Mini Mental State Examination (MMSE), SCOPA-Cognitive (SCOPA-Cog), Non-Motor Symptoms Questionnaire and PD-CRS.	• The average scores on PD-CRS was: 60.9 ± 16.5 (subcortical items), 27.9 ± 4.4 (cortical items) and 88.7 ± 19.8 (total PD-CRS score). • There was no ceiling effect or floor effect. • The Cronbach alpha was 0.85; • There was a high correlation with the MMSE and Scopa-Cog (rS = 0.53 and 0.77). • The PD-CRS scores were significantly lower in older patients, with low education level, and increased severity of cognitive symptoms as assessed with CISI-PD.	• The small sample size of the PD patients could limit the factorial analysis and the analysis of different subgroups of cognitive impairment. • No formal classification of cognitive impairment was used (e.g. MDS criteria). • Unclear risk of bias regarding the blinding of assessors
Fernandez-Bobadilla 2013 [57]	To examine the sensitivity to longitudinal change of the PD-CRS in non-demented patients with PD, and to provide a cutoff value of the scale for differentiating cognitively intact patients and PD-MCI patients.	The discriminative power of the PD-CRS for PD-MCI was examined in a sample of 234 patients (145 in the PD- normal cognition group; 89 in the PD-MCI group) and in a control group of 98 healthy individuals. Investigators who were blinded to PD-CRS scores classified a cohort of prospectively recruited, nondemented patients into a PD with normal cognition (PD-NC) group and a PD-MCI group using Clinical Dementia Rating (CDR) and the Mattis Dementia Rating Scale-2 (MDRS-2). In addition, the global cognitive status was assessed with the Cognitive	• In the binary logistic regression (forward; conditional) analysis of factors that had a significant correlation as covariates, the PD-CRS total score ($p < 0.001$; odds ratio, 0.92; 95% confidence interval, 0.89–0.94) and age ($p = 0.010$; odds ratio, 1.06; 95% confidence interval, 1.01–1.11) were identified as the best variables to independently differentiate PD-MCI from PD-NC. The other factors (education, PD evolution, H&Y staging, UPDRS-III, and depression score) were not predictive of PD-MCI. • The AUC analysis (AUC = 0.85;	• The lack of comprehensive neuropsychological assessment to classify the whole sample according to the Level II MDS criteria • Not all patients who were recruited for the cutoff study participated in the longitudinal study. • It is unclear if a consecutive or random sample of patients was recruited

		Impairment item in part I of the MDS-UPDRS (MDS-UPDRS cog-I). Patients with PD who had MDRS-2 scores >123 were classified with either PD-NC (CDR score, 0; MDS-UPDRS cog-I score, 1; MDRS-2 subscores, no impairment, or PD-MCI (CDR score, 0.5; MDS-UPDRS cog-I score, 1–2; MDRS-2 score, impaired in at least 1 subscore).	95% IC, 0.80–0.90) indicated that a score 81 of 134 was the optimal cutoff point on the total score for the PD-CRS (sensitivity = 0.79; specificity = 0.80; PPV = 0.59; NPV = 0. 91). • A range of change from 10 to 13 points on the PD-CRS total score was indicative of clinically significant change. For the PD-MCI patients, a decrease of 14 points indicated clinical worsening, and an increase of 11 points was the minimum change for a relevant improvement in the patient's clinical status.	
Fernández-Bobadilla 2017 [59]	To develop an alternative form (AF) of PD-CRS, that could minimize practice effects associated with repeated testing.	The study assessed a prospective sample of 75 non-demented PD patients (NC = 50; PD-MCI = 25) using both tools, PD-CRS and PD-CRS/AF, administered on 2 consecutive days, in a randomized order. Five cognitive domains (attention and working memory, language, memory, visuospatial skills and executive functions) were examined by a trained neuropsychologist using standardized and recommended neuropsychological measures: Trail Making Test Forms A and B, the backward digit span task, phonetic and semantic verbal fluency, the Free and Cued Selective Reminding Test, the Rey–Osterrieth complex figure test, the Boston Naming Test, the Judgment of Line Orientation, and the number location subtest of the Visual Object and Space	• ROC curve analysis for the PD-CRS showed that the optimal cutoff for PD-MCI was 81 (sensitivity 0.94; specificity 0.73) with AUC of 0.91 (95% CI, 0.840–0.982). • ROC curve analysis indicated for PD-CRS/AF similar results. The total score of 81 presented a sensitivity of 0.92 and a specificity of 0.73, with an AUC of 0.887 (95% CI, 0.807–0.967). • There was a strong correlation between the two scales for the total score and separate sub-scores. The data suggested that the two versions can be used in either order. • The study did not find any practice effects as a result of administering two similar instruments over a short period of	• The relatively small sample size of the study. • The authors tested only a sample of non-demented PD subjects. • There is no data regarding the scale's performance in PDD • It is unclear if a consecutive or random sample of patients was recruited

		Perception Battery).	time.	
Koevoets 2018 [61]	To evaluate the accuracy of the PD-CRS and the MDRS-2for detecting PD-MCI.	The study included 75 healthy subjects and 125 PD patients who were candidates for DBS.<br>Data from healthy subjects were used to correct for demographic influences.<br>The authors compared the accuracy of the two instruments using ROC analysis.<br>The gold standard was level II diagnosis of PD-MCI according to consensus criteria of the International Parkinson and Movement Disorder Society.<br>Level II diagnosis of PD-MCI was established if either the patient or an informant reported cognitive decline, and if the patient obtained abnormal scores (more than 1.0 SD below the demographically corrected mean) on at least two tests in one domain or in two or more separate domains.	• 27% of the patients had PD-MCI; none of the patients met PDD criteria.<br>• The PD-MCI patients performed significantly worse than the PD patients with normal cognition on the PD-CRS and the MDRS-2, both with and without demographic correction (*t*-test, all $p < 0.001$)<br>• Education level, age and sex correlated with the PD-CRS, but only age correlated with the MDRS-2.<br>• AUCs for raw scores of PD-CRS and MDRS-2 were 0.83 and 0.81, respectively. At the optimal cut-off for the PD-CRS (based on Youden index) was 101/102 (sensitivity 0.88 and specificity 0.64). For the MDRS-2 (139/140) sensitivity and specificity were 0.68 and 0.79, respectively.<br>• AUCs for demographically corrected scores of PD-CRS and for age-corrected scores of MDRS-2 were 0.80 and 0.78, respectively.<br>• Both cognitive screening tools were found to be suitable for distinguishing PD-MCI patients from cognitively intact PD patients.	• The authors relied on the control subjects' judgments whether or not they were cognitively healthy. Therefore, it is possible that not all control subjects were cognitively intact; some control subjects scored below the cut-off for PD-MCI.<br>• The study investigated a sample of patients who were candidates for DBS screening. Therefore, this is a biased sample of PD patients as DBS patients are generally younger than the average PD patient.<br>• The PD-MCI sample is rather small, with only 34 PD-MCI cases. A large sample is needed to show statistically significant differences between diagnostic instruments.<br>• The approach used to diagnose PD-MCI might need some improvement. It has not yet been established which neuropsychological tests are optimal for determining decline in each cognitive domain.<br>• The authors used the cut-off of 1.0 SD below average as definition of abnormal performance. Although this is in line with the cut-off for diagnosing PD-MCI proposed by the MDS task force, the presence of PD-MCI could be overestimated. However, the study found a mean prevalence of 27.2% of PD-MCI, which is comparable to what has been reported in the literature (range: 19%-38%).<br>• There was not always an informant to

				provide information on the patient's cognitive decline.<br>• The time interval between cognitive tests is unclear
Serrano-Duenas 2016 [59]	To investigate the measurement properties of the PD-CRS compared with Movement Disorders Society Task Force criteria for the diagnosis of dementia in patients with PD.	The sample consisted of 223 The study included 223 patients who were diagnosed in accordance with the United Kingdom Parkinson's Disease Society Brain Bank who were assessed with both the MDS-TF and the PD-CRS criteria (in addition to other instruments). All patients were evaluated first using the Level I MDS-TF criteria which include the MMSE.	• The internal consistency of PD-CRS was shown to be adequate, with a λ value of 0.821; the λ value rose to 0.831 with the elimination of the Action Verbal Fluency item.<br>• The test-retest correspondence was 0.81<br>• A floor effect was found in 4 of the items (Sustained Attention, Working Memory, Immediate Verbal Memory, and Alternating Verbal Fluency), and 1 item showed a ceiling effect (Clock Copying).<br>• The PD-CRS adequately discriminated patients with and without dementia (Kruskal-Wallis; $p \leq 0.000$).<br>• The AUC was 0.899. With a cutoff score of 62 (from a possible score of 134), the scale achieved 94% sensitivity and 99% specificity.<br>• The mean time for evaluation with the PD-CRS was 19.72 minutes (range, 13.89–22.67 minutes).<br>• The maximum convergent validity of the PD-CRS was 0.763 with the MMSE. Overall, the convergence values varied between 0.3 and 0.59.	• The reference standard was an MDS Level I cognitive assessment<br>• The time interval between the cognitive tests is unclear
Samat 2017 [60]	To determine the	The authors assessed the cognitive	• Using the MoCA test, 26 (56.5%)	• The small sample size may limit the

	prevalence of MCI among PD patients using MoCA and PDCRS (Level I MDS criteria). To determine the prevalence of executive dysfunction using the CTMT and correlate the presence of cognitive impairment with measurable biomarkers such as ApoE4 and plasma a-synuclein.	functions in 46 PD patients with MoCA, PD-CRS and CTMT test.	patients had PD-MCI and 20 (43.5%) patients had normal cognitive function. • Based on the PDCRS, only 36.9% of the patients were in the PD-MCI category, 39.2% had normal cognition and the remaining 23.9% were labeled as PDD. • 11 out of 26 patients (42.3%) with PD-MCI on MoCA were reclassified as PDD using the PDCRS. In addition, 5 (25%) out of 20 patients with normal cognition on MoCA were re-categorized as PD-MCI using the PD-CRS. • 3 of 26 patients with PD-MCI on MoCA were classified as normal cognition on the PD-CRS, giving a false positive value for MoCA of 11.5%. • The PD-CRS total score was significantly different between both groups, with normal cognition and PD-MCI ($p < 0.001$). • There were significant differences in the PD-CRS subcortical and cortical scores between the two groups (with intact cognition and PD-MCI). • Using bivariate analysis, there was a positive significant correlation between MoCA and PDCRS scores ($p = 0.01$).	results • The cognitive functions were assessed in accordance with the MDS Level I criteria; Level II criteria would have been preferred to confirm the presence of PD-MCI using more detailed neuropsychological testing. • It is unclear if a consecutive or random sample of patients was recruited • No data on the blinding of assessors • Incorporation bias
Tan 2020 [62]	To test the reliability and validity of a	The study investigated the cognitive abilities in 92 PD patients.	• The PD-CRS presented a high internal consistency	• The small sample of PDD patients might cause bias to some results, such as the high

	Chinese version of PD-CRS, establish cutoff scores for diagnosis of PDD and PD-MCI. To explore cognitive profiles of PD-MCI and PDD, and find cognitive deficits suggesting a transition from PD-MCI to PDD.	The patients were evaluated with PDCRS, MDRS and CDR. Based on CDR scores, the PD patients were divided into PD-NC, PD-MCI, and PDD subgroups; CDR = 0 in the PD-NC group, CDR = 0.5 in the PD-MCI group, and CDR ≥1 in the PDD group.	(Cronbach's Alpha = 0.840). • Intraclass Correlation coefficient (ICC) of test-retest reliability reached 0.906 (95% CI 0.860–0.935, $p < 0.001$). • ICC of inter-rater reliability was 0.899 (95% CI 0.848–0.933, $p < 0.001$). • PD-CRS presented a good concurrent validity with MDRS (ICC = 0.731, 95% CI 0.602–0.816). • In PD-MCI, all the frontal-subcortical items showed significant decrease, in comparison with the PD-NC group (p ≤ 0.001), but the instrument cortical items did not (confrontation naming p = 0.717, copying a clock p = 0.620). • In PDD, all the frontal-subcortical and instrumental-cortical functions showed significant decline, compared with the PD-NC group ($p \leq 0.001$). • The optimal cutoff value for diagnosis of PD-MCI was 80.5 (AUC: 0.803, 95% CI: 0.709–0.898, $p < 0.001$, sensitivity = 75.7%, specificity = 75.0%, PPV = 75.2%, and NPV = 75.5%) • The optimal cutoff for diagnosis of PDD was 73.5 (AUC: 0.984, 95% CI: 0.957–1.000, $p < 0.001$, sensitivity = 89.2%, specificity = 98.9%, PPV = 98.8%, and NPV = 90.1%). • Nonfloor effects were found for the total, subcortical, and cortical scores of the PD-CRS when	level of floor effects in PDD subgroups. • PD patients with high Beck Depression Inventory (BDI) scores which might act as a confounding factor for cognitive function test were not excluded. The study included 7 out of 44 PD-MCI patients (15.91%) and 4 out of 11 PDD patients (36.36%) who had BDI scores ≥20. • Nonetheless, the cognitive function was analyzed between BDI <20 and BDI ≥20 in PD-MCI and PDD subgroups separately and the results showed that PD-CRS total score and each item score have no significant difference between BDI <20 and BDI ≥20 scores in both PD-MCI and PDD subgroups. • It is unclear if a consecutive or random sample of patients was recruited • The data regarding the assessor's blinding for the reference standard is unclear • The reference standard are not the MDS Level II criteria

analyzed in all PD patients, specifically PD-NC and PD-MCI subgroup.

• In the PDD subgroup, items of immediate free-recall verbal memory, confrontation naming, sustained attention, working memory, alternating verbal fluencies, and delayed free-recall verbal memory showed floor effects, indicating that those cognitive functions were severely and commonly impaired in PDD patients.

• The ceiling effect was observed in confrontation naming (15.2%), clock drawing (32.6%), and copying a clock (72.8%) when analyzed in whole PD study group.

• Confrontation naming (21.6%), sustained attention (21.6%), clock drawing (54.1%) and copying a clock (86.5%) showed ceiling effects in the PD-NC subgroup.

• Only copying a clock showed the ceiling effect (20.5%) in the PD-MCI subgroup.

PD-MCI: Parkinson's disease mild cognitive impairment; PDD: Parkinson's disease dementia; PD-CRS: Parkinson's Disease-Cognitive Rating Scale; ROC: receiver operating characteristic; AUC: an area under the curve.
